# Medical student researchers in Colombia and associated factors with publication: a cross-sectional study

**DOI:** 10.1186/s12909-017-1087-9

**Published:** 2017-12-15

**Authors:** Francisco Javier Bonilla-Escobar, Juliana Bonilla-Velez, Daniel Tobón-García, Ana María Ángel-Isaza

**Affiliations:** 1 Department of Ophthalmology, University of Pittsburgh, UPMC Mercy Hospital - Eye Center, 1400 Locust Street, Suite 3013, Pittsburgh, PA 15219 USA; 2 Fundación Somos Ciencia al Servicio de la Comunidad, SCISCO, Cali, Colombia; 3 Universidad del Valle, Grupo de Investigación en Rehabilitación de la Universidad del Valle, GIRUV, Cali, Colombia; 4 Resident Physician, Department of Otolaryngology, Head and Neck Surgery, University of Arkansas for Medical Sciences, Little Rock, AR USA; 5 Andes University, Bogotá, Colombia; 6 Universidad Libre de Cali, Cali, Colombia

**Keywords:** Students, Medical, Education, Research, Scientists for health and research for development (MeSH)

## Abstract

**Background:**

Gaps between evidence-based research and clinical-public health practice have been evident for decades. One of the aims of medical student research is to close this gap. Accordingly, evaluating individual and environmental factors that influence participation of medical students in research are needed to understand and identify potential targets for action. This study aims to identify characteristics of medical student researchers in Colombia and the associated factors with scientific publications.

**Methods:**

A cross-sectional study of Colombian medical students involved in research using a validated, self-administered, online survey. The survey was distributed through the Colombian Association of Medical Students’ Associations (ASCEMCOL). Data sets were analyzed using descriptive and summary statistics. Bivariate analysis and a multiple logistic regression model were conducted to identify predictors of scientific publications.

**Results:**

A total of 133 responses were analyzed from students at 12 Colombian cities and 20 higher-education institutions. Although 94% of responders had at least one research proposal, only 57% had completed a project, and 17% had published their findings. Barriers for undertaking research included time restrictions and a lack of mentorship. Motivational factors included opportunity to publish findings and good mentorship. Students planning to do a specialization (OR = 3.25; 95% Confidence interval [CI] = 1.27–8.30), innovators (OR = 3.52; 95%CI = 1.30–9.52) and committed (OR = 3.39; 95%CI = 1.02–11.29), those who had previously published their findings (OR 9.13 IC95% 2.57–32.48), and were further in their medical education (OR 2.26 IC95% 1.01–5.07), were more likely to publish scientific papers.

**Conclusions:**

Our findings describe medical students understanding of the process of conducting research in Colombia. Although there appears to be motivation to participate in research, very few students achieve publication. Barriers such as time constraints and mentorship seem to play a critical role. This highlights opportunities where barriers to research can be overcome in medical school and other levels.

**Electronic supplementary material:**

The online version of this article (10.1186/s12909-017-1087-9) contains supplementary material, which is available to authorized users.

## Background

Under the paradigm of Evidence-based medicine and evidence-based public health (EBM; EBPH) research plays a central role in determining best practices in patient care. Unfortunately, there is a global deficit of medical researchers despite an increasing demand for them [1, 2]. One of the limitations has been the lack of physician engagement in research early in their careers, for example as medical students [3–8]. To address these phenomenon, medical education has incorporated classes on research methodology and epidemiology in their curriculum to prepare their graduates for clinical care, and some universities require the completion of a research project prior to graduation. Although education trends support that an effective educational tool is learning by practice, medical student involvement in research overall remains an activity of a minority of students and they often lack support mechanisms for their initiatives. This is especially true in low- and middle-income countries where intellectual, structural and financial support mechanisms are often lacking. Other barriers may include lack of interest or poor attitude towards research. Some studies have mentioned the factors that motivate and demotivate young people to undertake research [3–5, 9–13]. Motivators that have been described include: good mentorship and enhancing visibility through indexed publications [5–7, 14]. Factors that make it difficult for or demotivate young people to undertake research include high costs, little financial return in the long run (in comparison to clinical practice peers) and lengthened training. Individual factors influencing medical student engagement in scientific research activities are students with a high grade point average (GPA) and personality dimensions “openness to experience”, “conscientiousness”, and “extraversion” [15].

In Colombia, according to the Administrative Department of Science, Technology and Innovation of Colombia (Colciencias), there are over eight thousand researchers who are part of 5869 research groups, but health science research groups are the least common ranking in the bottom three for number of researchers in the field [16]. Moreover, from the 57 medical schools in the country, there is no published information on medical student demographics or their involvement in research. Research is the mainstay of science; without it there cannot be advances in the management of disease. Therefore, it is imperative to generate strategies to enhance medical student appreciation of research during their training and promote its integration to their future careers as an instrument to generate knowledge and provide support to the community [17]. As a first step towards this goal, this study aims to identify characteristics of medical students who have successfully completed research projects; their environment, barriers to research and motivational factors; with the hope to use this information to direct further research and interventions that will strengthen research capacities among physicians in the country and contribute to medical practice.

## Methods

A cross-sectional study based on a self-administered survey was conducted among Colombian medical students to identify factors associated with an active involvement of medical students in research. There is no a unified database of medical students enrolled in medical school in Colombia, nor of their participation in research. Therefore, a convenience sample was used based on existing scientific associations of medical students. The survey was distributed to all the medical student members of each Medical Students’ Scientific Societies (MSSS) that participate in the Colombian Association of Medical Students’ Associations (ASCEMCOL, www.ascemcol.org), a national association founded in 1989 which has historically accrued students with motivation for research. ASCEMCOL unites MSSS from 30 higher-education institutions (HEI) around the country and therefore would offer good representation of medical students around the country, especially of those who are participating in research. The inclusion criteria were medical students who were actively involved in a MSSS, had at least one ongoing research project, and had passed their first semester of studies. Being involved in a MSSS implies that the student is enrolled in a medical school of a certified HEI (recognized and entitled by the Colombian government as having undergraduate, graduate and postgraduate programs). Medical students pursuing more than one simultaneous degree were excluded from the study to guarantee homogeneity of the population under study.

The survey was created as a Google® Drive questionnaire which was sent electronically to all potential participants using ASCEMCOL’s database with a cover letter inviting them to participate in the study. The email was sent on October and responses were received until December 2009. The questionnaire used to identify the characteristics of medical students doing research was previously validated and published elsewhere. [18] The questionnaire was composed of sections that included (A) socio-demographic and financial aspects, (B) research influences, (C) research affiliations, (D) research productivity, (E) extracurricular activities, (F) research motivations, (G) career-research relations, and (H) research benefits and professional plans, (I) personal attributes (measured on a Likert scale from 1 to 5). For research productivity only books, book chapters, letters to editors, case reports, systematic reviews, and original papers were considered as scientific publications. Other publications were counted as non-scientific. For a full description of the survey utilized, please see Additional file: 1.

For data analysis the database was initially subjected to strict quality control through an exploratory analysis. Extreme values were identified and confirmed (i.e. the number of publications was verified with the name of each publication). Data was analyzed using summary statistics and dispersion analysis followed by inferential statistics. A Chi-square or Fisher’s exact test was used for comparison of categorical variables and a t-test or Wilcoxon test was used for quantitative variables, depending on the assumptions of each specific test. A bivariate analysis was conducted initially to identify potential associations with the variable “publications” (having published a scientific paper) with a statistical significance of <0.20, according to Hosmer-Lemeshow [19]. Variables associated with publications were included in a multiple logistic regression model using “publications” as the dependent variable. Additionally, age, sex, type of university (public vs. private), having parents who are researchers, being part of a research group recognized by Colciencias, and having presented research findings at a scientific event, were included variables as potential confounders. The final model was estimated with the maximum likelihood test. All statistical analyzes were done using Stata 13® (Stata Corp, TX, USA).

## Results

A total of 159 responses from medical students were received; 26 were excluded because the respondents were not actively involved in a research project or were enrolled in two careers, for a total of 133 accepted responses. The socio-demographic characteristics of participants are displayed in Table 1 *.* Participants were from 12 Colombian cities and 20 HEI, of which 12 (60%) were public institutions. Forty one percent of students were studying medicine in Cali, 16% in Pereira and 9.7% in Tunja, among other cities with less participation. Females represented 51% of participants. Out of the 133 participants, 77% were enrolled in a public HEI and 17% had a job in addition to their medical training, which in most instances, was not related to the medical field. Most students were involved with a research group (77%) and almost 90% of them joined the group during their first three years of studies. A minority of students were part of a research group recognized by the Colciencias (26%). The motivators for joining research groups included personal affinities with the research topic (66%) and a desire to acquire research methodology skills (26%); prestige and networking as motivators were not highly valued (6% and 3%, respectively).

**Table 1:** Socio Economic Characteristics of Medical Student Researchers

Characteristic	Frequency	Percentage
Civil status		
Single	129	96.99%
Married	1	0.75%
Cohabitation	3	2.26%
Gender		
Male	65	48.87%
Female	68	51.13%
Type of University		
Public	103	77.44%
Private	30	22.56%
Year of studies		
1–2	30	22.56%
3–4	62	46.62%
5–6	41	30.83%
Work on the side		
Yes	22	16.54%
No	111	83.46%

Of respondents, 94% have at least one research proposal, 43% have two or more research protocols with only 4% having four or more proposals. The 6% with no research proposals had at least one finished project. When inquiring about finished research projects data shows that 43% have not finished a research project, 38% have one finished project, and 20% have at least two.

Despite all students being involved in research, 6% have at least one publication as a first author and 1.3% have two or more, while 12% have at least one publication as a co-author and 1.3% have two or more. Overall, 29 (22%) students have a publication and 19 publications were cited as evidence; this is due to the fact that one publication is shared by several students. From the 29 students who have published, 19 were as co-authors and 10 as first authors. From the 19 published papers, 12 were originals, 3 of them case reports, 2 systematic reviews, and 2 letters to the editor. Regarding the journal where papers were published, 10 were national and nine were international journals. In addition, 10 (52.6%) of the articles were indexed in MEDLINE. Medical students also reported participation in other research related activities with 46% of students having been part of a scientific event organizing committee. Thirty-six percent of students have had an oral presentation at a scientific event; 23% of these had presented once and 13% had presented twice or more.

Factors that influence medical students participating in research are illustrated in Table 2 *.* The principal motivations for participation in research were the perceived interest to publish (47%), curiosity (31%) and to a lesser extent because they were advised to do so (14%). Influences by professors (34%) and classes on research methodology (22%) were most important in stimulating interest in research. In terms of role models as motivators, 83% claim that none of their parents do research, 71% reported having a physician as a role model with 62% of them stating that their role model was also a researcher (58.5% of the total). Participants also identified the factors that make it most difficult to pursue research. Time restrictions was thought to be the most important factor for 71% of respondents, followed by lack of meaningful mentorship (30%) and scarce financial resources (20%), among others. On another note, 16% of responders had received any kind of incentive by their university for doing research. Incentives included funding (33%), public recognition of merits (24%), and a diploma (19%), among others. Funding was mainly monetary to cover travel expenses for scientific events. One respondent described having received a scholarship that covered the entire tuition fees for the rest of medical studies.

**Table 2:** Situations around Medical Students’ Engagement in Research Activities

Characteristic	Frequency/Central Tendency Measure
What got them to engage in research [n (%)]	
Area of study	25 (18.80)
To acquire research methodology skills	9 (6.77)
To establish contacts	2 (1.50)
Prestige	4 (3.01)
No answer	93 (69.92)
Motivators [Mean (SD)]	
Prestige	3 (1.43)
To publish	4 (1.37)
Attend events	3 (1.39)
Better chances for residency	4 (1.46)
To network	3 (1.34)
To stay updated	4 (1.32)
To meet new people	3 (1.4)
Barriers to do research - Demotivators [n (%)]^a^	
Lack of access to research	1 (0.75)
Scarce financial resources	26 (19.55)
Lack of quality mentorship	40 (30.08)
Difficulty in accessing the study population	8 (6.02)
Lack of time	95 (71.43)
Little support from university authorities	14 (10.53)
Lack of curricular flexibility	
Adjectives [mean (SD)]	
Honest & Fair	4.58 (0.61
Hardworking	4.51 (0.64)
Ethical	4.42 (0.77)
Intelligent	4.37 (0.71)
Persistent	4.26 (0.88)
Committed	4.23 (0.74)
Sociable	3.93 (1.13)
Rigorous	3.82 (1.01)
Personality [n (%)]	
Introvert	58 (43.61%)
Extrovert	75 (56.39%)

^a^ *multiple-choice question*

It is noteworthy that only 7% of participants perceive that doing research had a negative effect on their medical school performance, but 21% believed they could perform better (obtain higher GPAs) if they were not involved in research. Of importance, 75% of participants had a GPA above 3.7 and only 10% were below 3.5 on a scale from 0 to 5 where 3.0 is the passing grade. Following up on medical school performance, 32% of participants had failed and repeated classes and, of those, 42% had failed and repeated at least two classes. Moreover, 6% identify doing research as the reason for having failed a class.

When asked about other aspects of medical student researchers’ lives, the study found that 85% are engaged in extracurricular activities such as student councils and other student-led groups, sports and reading non-medical literature. When examining the participants’ self-assigned adjectives, on a scale from 1 to 5, the most identified ones were: honest/fair (mean 4.58 ± 0.61) and hardworking (4.51 ± 0.64). See Table 2 *.* Furthermore, 84% of responders described an intention to keep on pursuing research in the future; 16% were uncertain and 0.65% did not intend on pursuing further research.

In bivariate analysis, correlation was discovered between “publications” and other characteristics of the participants. For instance, “age” (*p* = 0.003) and “year of studies” (*p* = 0.0001) were associated with having published scientific papers (Table 3). Lastly, multiple logistic regression analysis shows that the opportunity to publish among medical students doing research is 0.22 (95% Confidence interval [CI] = 0.14–0.34, *p* < 0.001). It was found that participants who consider themselves as “innovators” have an opportunity 3.5 times greater to publish scientific findings than those who do not. Furthermore, “committed” participants had an opportunity to publish scientific papers of 3.38 compared to those who do not. Participants with more than one finished research project had a probability 9.13 times higher of publishing papers than those who have none (Table 4). Difference of log-likelihood ratio of the intercept only (−62.786) compared to the full model (−28.825) was statistically significant (Chi-square = 67.9, *p* = 0.0001, McFadden’s Adjusted R^2^ = 0.350).

**Table 3:** Characteristics of Medical Student Researchers based on Publications

Characteristic	Publications		p-value
	Yes	No	
Age [mean (SD)]	22.70 (2.36)	21.11 (2.36)	0.003
Sex [Freq (%)]			0.9
Male	12 (50)	53 (48.62)	
Female	12 (50)	56 (51.38)	
Year of study [Mean (SD)]	8.83	6.55	<0.0001
Type of University [Freq (%)]			0.4
Public	20 (83.33)	83 (83)	
Private	4 (16.67)	26 (23.85)	
Parents-as-researchers [Freq (%)]	4 (16.66)	18 (16.51)	0.9
In research Group in Colciencias [Freq (%)]	9 (37.5)	26 (23.85)	0.2
Number of finished research projects [Mean (SD)]	2.20 (1.47)	0.59 (0.69)	<0.0001
Speaker at scientific events [Freq (%)]	15 (62.50)	33 (30.27)	0.001
Motivations/Qualities [mean (SD)]			
Interest in networking	3.58 (1.24)	2.92 (1.41)	0.03
Prestige and recognition as motivator	3.83 (1.46)	3.26 (1.41)	0.05
Committed person	4.62 (0.57)	4.14 (0.75)	0.003
Innovative person	4.29 (0.75)	3.75 (0.93)	0.006
Persistent person	4.62 (0.77)	4.18 (0.89)	0.009
Rigorous person	4.25 (0.73)	3.73 (1.05)	0.03
To enter residency	4.62 (0.92)	3.97 (1.54)	0.06

**Table 4:** Multiple Logistic Regression Analysis of Publications and Medical Student Researchers’ Characteristics

CCharacteristic	OR^a^	95% CI^b^	p-value
Research as support for residency application /admission	3.25	1.27–8.30	0.01
Considering himself/herself innovator	3.52	1.30–9.52	0.01
Year of studies	2.26	1.01–5.07	0.05
Considering himself/herself committed	3.39	1.02–11.29	0.05
Number of finished research projects	9.13	2.57–32.48	0.001

^a^Analysis adjusted by age, sex, type of university, parents as researchers, research group in Colciencias, and being a speaker at a scientific conference

^b^ 95% Confidence interval

## Discussion

Medical schools are one of the most important factors in the formation of medical researchers [20]. We report results of a survey to medical student researchers in Colombia. Factors that students identified as motivators for research included interest in publishing and curiosity as well as having strong role models, while the most important limiting factors were time restrictions and lack of mentorship. Some incentives for performing research included funding to support presenting their research and public recognition. We identify medical student researchers as active members of their society with over 85% of them participating in other extracurricular activities. Students with self-perceived traits as innovator and committed had greater probability of having publications, and having had a publication increased probability of further publishing papers by 9.13. Importantly, 84% of students reported their intention to continue to do research in their future careers.

Mentorship has been identified to be crucial for pursuing a successful and satisfying academic career [8, 21–25]. The fact that 59% of the medical student researchers have a physician-scientist as a role model emphasizes the importance of promoting medical research, by example, as an exemplary career path. Other studies have highlighted the importance of meaningful mentoring for promoting medical research. Ley and Rosenberg have called upon physician-scientists to “stop being invisible” while quoting Michael J. Welsh, and urged them get in closer contact with students and residents and their patients [12]. Angel et al., also identified in their study population active in research that only 57% had a physician-scientist role model. Furthermore, a study carried out in a region of Saudi Arabia among residents showed that 93% lack research training and 73% lack supervisors; these are seen as important barriers to undertaking research [13]. The 30% of participants from the current study said the lack of adequate mentorship actually demotivates those already involved in research.

Considering that interest in an area of study was the most important aspect that got the students engaged in research and that having enthusiastic professors were one of the most influential factors, it is important to ensure that research training is delivered in a cross-sectional and interdisciplinary fashion [26–28]. Doing so would guarantee that critical thinking and research capabilities are present throughout the spectrum of medical and health professionals.

Time restriction has been identified as a significant barrier for research engagement, a perception not only correlated to the poor incorporation of research training programs, based on EBM and EBPH, in the medical curricula but also to the lack of incentives to do research [9, 10, 12]. A research focused career is known to require a lot of time in training and executing projects which often comes with high debts [12]. Nevertheless, some faculties seem to be coming up with alternatives to counter these barriers [10]. In our study, likewise, the perceived scarcity of financial resources came in third as a barrier for undertaking research.

It is important to note that incorporating EBM and EBPH training in medical and other health related curricula can enhance the visibility of a university or faculty at different levels, as explained by Cursiefen and Altunbas [7]. Colombian universities, and Colombia as a country, could benefit from this considering that the country came fifth in the area of scientific production in Latin America in 2012 [29]. With our finding of 84% of medical students performing research having an interest in continuing to do this in their future careers, it is imperative that medical schools and governmental policy continue to promote scientific efforts among medical students as this could contribute to strengthening scientific production and innovation nationally. Without the proper politics in effect to promote medical students’ interest in research, EBM and PHEB will be at risk [30, 31].

Scientific publications are the most tangible proof and tool for follow up on research activity; they represent the sharing of new knowledge that has the potential to benefit all of society [11]. Furthermore, publishing articles represents an activity that requires a great amount of effort. This study found that having publications is significantly associated with age and year of studies: as the medical students grow older (both in age and in their studies) the probability of having publications increases. Also, as the students invest more hours weekly on research, the likelihood of publishing papers enhances. All these variables are correlated with the fact that the more research proposals and finished research projects a student has, the more chances the student gets for publishing scientific papers. Some universities like Universidad Peruana Cayetano Heredia in Peru, have adjusted their thesis requirements to the ones often used by scientific journals; [32] also, other studies found that a thesis would be an acceptable pre-requisite for a Doctor of Medicine degree [22, 33, 34]. Although more evidence on this area is needed in order to make appropriate evidence-based decisions in medical curricula and postgraduate curricula, this strategy shows promising as it clearly relates to the opportunity to make scientific publications.

Furthermore, the multivariate analysis shows how participants who think of themselves as “innovators” or “committed” generally benefit from more opportunities to publish. Also worthy of mention is that given the difficulties of accessing residency programs among Colombian medical doctors, our participants’ belief of published papers as adjuvant for residency application is associated with a higher opportunity for publishing scientific findings. These results can support faculty authorities and the scientific community and activists in targeting the factors that facilitate or hinder culmination and appropriate dissemination of research findings. The study that inquired about student engagement in scientific research in Portugal [15] gives us an understanding of the characteristics that are related with scientific engagement in that specific population. Nevertheless, it falls short in identifying potential areas where student advocates and university decision-making authorities can act to counter the issue. The present study not only identifies individual and contextual characteristics that need to be considered in medical training but gives a glance of some alternative solutions. Furthermore, the indicator of publications allows stakeholders to make follow-up assessments of meaningful student engagement in scientific research [35].

In view of all the factors that hinder medical students undertaking research and the factors that demotivate them, it is of high importance to note that 16% of medical student researchers are unsure about continuing to pursue research in the future. This figure is likely to be the result of the timid role EBM and EBPH has played in medical schools and shows the vicious cycle that recreates and broadens the gap between medical and public health practice and best-available evidence.

The study limitations include the sample size and the sampling method. This study did not calculate sample size as there is no information about the number of medical students in the country, but with a confidence of 95% and the number of respondents who fulfilled the inclusion criteria (*n* = 133), an error of 8.5% was calculated. Thus, it can be suggested that the sample size is acceptable for the obtained conclusions. Additionally, it is important to note that the 133 participants do not represent the totality of medical student researchers in Colombia. ASCEMCOL was present, at the moment of the survey, in 12 Colombian cities and had a total of 17 MSSS. Medical students’ associations are in constant generational change and its activities are, for the most part, a reflection of single or small-group initiatives. Considering this, it is relevant to mention that the most active MSSS at the time of the survey were those from Cali, Pereira, Tunja, and Cartagena. This fact explains why there were much more respondents from those cities. However, there is very limited evidence on medical education in the country, let alone in research within medical education which make this paper of high relevance for the raising evidence. On another note, of the 57 medical faculties in the country, none require research work for graduation; additionally, considering that the proportion of Senior Researchers in Colombia is less than 1% according to Colciencias, [16] research stimuli or funding for this population has not changed over the last years. Therefore, we considered that even if the data was obtained few years ago is still valid. It is also important that similar studies with greater samples are conducted, especially linking the qualitative aspects of student engagement and the quantitative output indicators of that engagement such as those lead by the Collaborative Working Group for the Research of Human Resources for Health (Red-LIRHUS) [36, 37]. Moreover, it is relevant to assess policies and measures to counter the physician-scientist deficit.

## Conclusion

Our findings describe medical students’ perspective of the process of conducting research in Colombia and the outcomes of an organized group of them with regards of scientific publications. Although there appears to be motivation to participate in research, very few medical students achieve the goal of a publication. Barriers such as time constraints and lack of mentorship seem to play a critical role in the process of training future scientist. This highlights opportunities where barriers to research at medical training level can be overcome and potentially into other levels such as high school or graduate level.

It is paramount that student leaders, physician-scientists, public health practitioners, and the scientific community make the most of this momentum and actively bring EBM and EBPH to the classrooms, hospitals and communities where the means of health professionals finally meet the ends. Should there be stronger policies that include research-related activities in health-related under- and post-graduate programs in Colombia? This question needs to be extrapolated to other contexts to promote research.

## Additional file

Additional file 1: Questionnaire of medical students’ characterization and research perspectives. (DOCX 24 kb)

## Abbreviations

ASCEMCOL: Colombian association of medical students’ associations.
CI: Confidence interval
Colciencias: Administrative department of science, technology and innovation of Colombia
EBM: Evidence-based medicine
EBPH: Evidence-based public health
GPA: Grade point average
HEI: Higher-education institutions
MSSS: Medical students’ scientific societies
Red-LIRHUS: Collaborative Working group for the research of human resources for health
