# Supplementary material for: Medical student researchers in Colombia and associated factors with publication: a cross-sectional study
Source: BMC Med Educ. 2017 Dec 15;17:254. doi: 10.1186/s12909-017-1087-9 (PMC5732498; doi:10.1186/s12909-017-1087-9)
Supplement: Additional file 1: — Questionnaire of medical students’ characterization and research perspectives. (DOCX 24 kb) [file 12909_2017_1087_MOESM1_ESM.docx]

**SURVEY ON CHARACTERIZATION OF MEDICAL STUDENT RESEARCHERS IN COLOMBIA**

**A. Socio-Demographic and Financial Aspects**

Date:

City:

State:

Age (in years):

Gender:

a. Male

b. Female

Marital Status:

a. Single

b. Married

c. In free union

d. Widow

e. Divorced

Religion:

a. Catholic

b. Christian

c. None

d. Other, which?

Socioeconomic level: By the location where you reside: 1___ 2___ 3___ 4___ 5___ 6___

University: __________ Public___ Private___

Semester:

**B. Research Influences**

1. Is either of your parents involved in research?

a. Mom

b. Dad

c. Both

d. None

2. Is either of your parents a physician?

a. Yes___ Mom__ Dad___ Both__

b. No

3. Is any of your family members a researcher?

a. Uncle/Aunt

b. Cousin

c. Grandparent

d. Other

e. None

4. Do you have a role model in Medicine?

a. Yes And they are a physician researcher___ They are a physician but dont do research___

b. No

**C. Research Affiliations**

5. Are you part of a student research group?

a. Yes, Name of the group:________

b. No

If no, please proceed to question 8

6. What semester were you in when you joined the student research group? _______

7. What motivated you to join? (Select one):

a. Curiosity

b. By recommendation

c. To travel to meetings

d. Incentives by the University to become involved

e. To meet people

f. To publish

g. For prestige

h. Other. Which?_________

8. Are you part of a research group recognized by the Administrative Department of Science, Technology and Innovation of Colombia - Colciencias?

a. Yes

b. No

If you answered no, proceed to question 12

9. In what semester did you join the research group recognized by Colciencias? ______

10. What motivated you to join? (Select one)

a. The topic/ research focus

b. or networking

c. By obligation, in my University you can only do research in a group recognized by Colciencias.

d. Payment for doing research

e. Its a good resource to learn research skills

f. Because it easier to do research as the projects are streamlined.

g. Prestige

h. Other, which? __________

11. What is the area of the research group recognized by Colciencias? (For example Ophthalmology, Surgery, etc.) ____________

**D. Research Productivity**

12. How many research projects are you working on?

a. 0

b. 1

c. 2

d. 3

e. 4

f. 5 or more

13. How many research projects are you in the process of executing?

a. 0

b. 1

c. 2

d. 3

e. 4

f. 5 or more

14. How many publications are you a co-author on?

a. 0

b. 1

c. 2

d. 3

e. 4

f. 5 or more

If you are a co-author, please describe:

Title, Journal, Year of Publication, Volume, Issue

15. How many publications do you have as first author?

a. 0

b. 1

c. 2

d. 3

e. 4

f. 5 or more

If you are a first author, please describe:

Title, Journal, Year of Publication, Volume, Issue

16. Have you published any of these type of publications?

a. Letter to the Editor

b. Review

c. Case Report

d. Art or history essay

e. Book chapter

f. Book

g. Meeting summaries

h. None of the above

If you have any of these type of publications, please describe:

Title, Journal, Year of Publication, Volume, Issue

17. Are you planning to publish all your ongoing research projects?

a. Yes

b. No

18. When seeking publication, do you submit to indexed journals?

a. Yes

b. No

c. I don’t know what that is

19. When seeking publications, do you submit to a national or international journal?

a. National

b. International

c. Either

20. Have you organized a research event?

a. Yes Which one?______

b. No

21. In how many meetings have you presented a research project (in planning phase) or a completed project (with results and conclusions)?

a. 0

b. 1

c. 2

d. 3

b. 4-6

c. 7-9

d. 10-12

e. >12

22. Would you say your research work is:

a. All in the same area/specialty

b. Almost all are in the same area/specialty

c. Are in different areas/specialties

d. I only have one

e. Other, what?______

23. What motivates you to do research? (Please score from 1 to 5 from what motivates you the most to least)

Prestige __

Publications __

Attendance to Meetings and Events __

Acceptance to Residency__

Networking __

Be updated on Medicine__

Meet new People__

**E. Extracurricular Activities**

24. Do you do any other extracurricular activity outside of research?

a. Yes

b. No

If you answered no, go to question 26

25. What extracurricular activity do you practice? (Select all that apply)

a. Sports

b. Gym

c. Student Council or other student-groups

d. Read (Other than Medicine)

e. Other, which? ___________

26.You would say about sports that:

a. You are passionate about sports

b. don’t want to know anything about it

c. You are indifferent to sports

d. You like to watch sports but not practice any

e. You would like to have more free time to do sports

27. About soccer you:

a. Enjoy playing soccer

b. Are not interested

c. Play occasionally with friends

d. Enjoy watching matches

e. Other, which?_______________

28. About painting you:

a. Like and admire it, but its not your thing

b. Don’tlike it and don’t want to know anything about it

d. Enjoy it and are good at painting.

e. You enjoy it and paint occassionally

29. What music genre do you prefer? __________________________________

30. Do you have a job?

a. Yes What is your job?________

b. No

31. Before medical school, did you study another profession or had another career?

a. Yes

b. No

If you answer no, proceed to question 33

32. The title obtained is: _______________

**F. Research Motivations**

33. On average, how many hours a week do you dedicate to research?

a. 1-2 hours

b. 3-4 hours

c. 5-6 hours

d. 7-8 hours

e. 9-10 hours

f. 10 -15 hours

g. 15- 20 hours

h. >20 hours

34. What do you think influenced you the most for to do research?

a. Friends

b. Family

c. Professors

d. Research methodology classes/courses

e. Advantages offered by the University to those who participate in research

f. Other, what? _____

35. What is the greatest difficulty a medical student has to do research? (Select one)

a. Poor guidance

b. Limitations in time available to do research

c. Limited curricular flexibility

d. Poor support from the administration

e. Poor support from professors

f. Other, what?___________

36. What is the factor that is most demotivating to a student performing research? (Select one)

a. Poor access to the population under study

c. Poor guidance

d. Limitations in time available to do research

e. Lack of support from friends

f. Lack of support from professors

g. Lack of support from the administration

h. Lack of financial support

i. Other, what? ___________

**G. Career-Research Relations**

37. Do you consider that being a student research negatively impacts your professional career?

a. Yes

b. No

c. I don’t know

38. Do you consider that you would be able to get better grades if you didn’t do research?

a. Yes

b. No

c. I don’t know

39. What is your approximate grade point average?

a. 3.0

b. 3.1

c. 3.2

d. 3.3

e. 3.4

f. 3.5

g. 3.6

h. 3.7

i. 3.8

j. 3.9

k. 4.0

l. 4.1

m. >4.1

40. Have you had to repeat a subject/course?

a. Yes

b. No

If not, proceed to question 44

41. How many subjects/courses did you have to repeat?

a. 1

b.2

c. 3

d. >3

42. What subjects/courses did you have to repeat?_____________

43. Do you consider you failed the subject/course because of your participation in research?

a. Yes

b. No

c. I don’t know

**H. Research Benefits and Professional Plans**

44. Have you received any incentives from your University for participating in research?

a. Yes

b. No

If your answer is no, proceed to question 46

45. What incentives did you receive? (Can mark more than one)

a. Full scholarship for a semester

b. Half scholarship for a semester

c. Discounted tuition

d. Public recognition

c. A diplomma

d. Other, What? ___

46. From the following adjectives, please score from 1 to 5 your perceived attributes, where 1 is what is most characteristic of you, and 5 the least.

Compromise __

Fair __

Have free time __

Emotional __

Ethical __

Creative __

Innovator __

Smart __

Persistent __

Rigurous __

Sociable __

Hard worker __

47. Do you consider you tend to be more:

a. Introvert

b. Extrovert

48. Do you plan to continue doing research after you graduate?

a. Yes

b. No

c. I don’t know

49. How do you see your practice in the future?

a. As a general practitioner

b. Specialist in a Medical Specialty

c. Specialist in a Surgical Specialty

d. Specialist in basic sciences

e. Specialist in public health

f. General practitioner with further training in epidemiology

g. Specialist in a Medical Specialty and epidemiology

h. Specialist in a Surgical Specialty and epidemiology

i. Other, what?___

50. In what do you plan to specialize in?

(For example: Cardiology, Endocrinnology, Gastroenterology, Nephrology, Pulmonology as sub-specialty training from Internal Medicine. Please answer for the broad specialty (internal medicine) rather than the subspecialty (Cardiology)).
